# Lupus Erythematosus Tumidus as a Distinct Uncommon Subtype of Cutaneous Lupus Erythematosus: A Case Report and Review

**DOI:** 10.7759/cureus.59140

**Published:** 2024-04-27

**Authors:** Charles Camisa, Veronica Papavero

**Affiliations:** 1 Medical Dermatology, Riverchase Dermatology, Fort Myers, USA; 2 Dermatopathology, Riverchase Dermatology, Fort Myers, USA

**Keywords:** lymphocytic infiltration of the skin, systemic lupus erythematosus, immunohistochemical markers, hydroxychloroquine treatment, photosensitivity disorders, polymorphic light eruption, chronic cutaneous lupus erythematosus, subacute cutaneous lupus erythematosus, discoid lupus erythematosus (dle), lupus erythematosus tumidus

## Abstract

Lupus erythematosus tumidus (LET) is an uncommon but distinct photosensitive subtype of cutaneous lupus erythematosus (CLE). It differs from discoid and subacute cutaneous lupus erythematosus (SCLE) clinically and pathologically. LET is marked by extreme photosensitivity and carries a much lower risk of progression to systemic disease. The differential diagnosis of LET includes polymorphic light eruption (PMLE) and Jessner’s lymphocytic infiltration of the skin (JLIS) because of subtle alterations in the histopathology and the paucity of immunopathologic markers in LET. We report herein a case of LET with positive immunoglobulin (Ig) deposits on direct immunofluorescence (DIF) testing. LET resolved completely with strict sun avoidance and treatment with topical corticosteroids, without the sequelae of atrophy, scarring, or dyspigmentation.

## Introduction

There are approximately 200 case reports and series of lupus erythematosus tumidus (LET) in the literature with an equal male-to-female incidence [1-3]. The clinical lesions of LET are reported as red- to violet-colored urticarial plaques, papules, or nodules with a smooth surface usually located in a sun-exposed area such as the head, neck, and trunk. There may be central clearing and slight scaling. The lesions tend to recur in the same locations, especially after sun exposure. LET is improved and may clear with topical corticosteroids and hydroxychloroquine therapy or spontaneously with strict sun avoidance. First-line treatment failures may also respond to methotrexate, thalidomide [1], or deucravacitinib [4].

Histopathology shows that the epidermis is normal with a mild thickening of the basement membrane. Mucin deposits are found in the papillary and reticular dermis. There are moderate superficial and deep perivascular and peri-adnexal lymphohistiocytic infiltrates [5]. The findings in Jessner’s lymphocytic infiltration of the skin (JLIS) are very similar with more dense perivascular lymphocytic infiltrates than LET. Abundant pools of mucin are typically not found in JLIS. Polymorphic light eruption (PMLE) shows variable epidermal spongiosis and more prominent edema in the papillary dermis compared to LET [6].

Antinuclear antibodies are positive at a low titer in 10%-20% of cases of LET according to the three largest case series [1-3]. Direct immunofluorescence (DIF) testing of LET lesional skin and autoantibody profiles is usually negative. In one case series of 40 patients with LET, all 40 cases gave negative DIF results [3]. In the same study, Sjögren’s syndrome A (SS-A) and Sjögren’s syndrome B (SS-B) antibodies were positive in two patients, double-stranded deoxyribonucleic acid (dsDNA) in one patient, and rheumatoid factor (RF) in two patients. U1 ribonucleoprotein (U1RNP), Smith, scleroderma-70 (Scl-70), and Jo-1 antibodies were negative in all 40. Interestingly, 30% of the patients had elevated C-reactive protein (CRP) levels, indicative of systemic inflammation.

Immunohistochemical analysis of LET cases [7], JLIS [8,9], and PMLE [9,10] has been performed: in LET, most of the cluster of differentiation (CD) 3 lymphocytes in infiltrates were CD4 helper T-cells with a CD4/CD8 ratio of 3:1 [7], whereas in JLIS and PMLE lesions, there was a clear predominance of CD8 cytotoxic T-cells in the majority of the patients tested [8,10]. Thus, the immunohistochemical labeling of CD3, CD4, and CD8 lymphocytes may be ordered on biopsies as an aid in differentiating LET from JLIS or PMLE.

## Case presentation

A 50-year-old female presented to the dermatology clinic for a rash on her right upper chest for about one month. She previously attended an urgent care center and was prescribed doxycycline 100 mg twice daily, oral diphenhydramine, and topical 1% hydrocortisone cream for possible Lyme disease. The patient had no recollection of a tick bite in the affected area.

The rash continued to enlarge despite this treatment. Upon examination, it consisted of a 3 cm erythematous plaque with central clearing. There was no evidence of scaling, vesicles, or pustules. The rest of her body skin was clear.

Western blot testing of serum was negative for immunoglobulin (Ig) M and IgG antibodies to Lyme disease. A complete metabolic panel gave normal results. A complete blood count with differential showed slight elevations of hemoglobin, hematocrit, and RBC indices (attributed to her history of smoking and alcohol intake). Lupus autoantibody profile gave negative results (Table 1).

**Table 1:** Pertinent laboratory results H, high; NEG, negative; MCV, mean corpuscular volume; MCH, mean corpuscular hemoglobin; MCHC, mean corpuscular hemoglobin concentration; RDW, red cell distribution width; ANA, antinuclear antibody; IFA, immunofluorescence assay; Sm, Smith; RNP, ribonucleoprotein; ds, double-stranded

Test	Patient Result	Normal range	Units
WBC count	8.9	3.8-10.8	Thousand per microliter
RBC count	4.63	3.8-5.1	Million per microliter
Hemoglobin	16.4, H	11.7-15.5	g/dL
Hematocrit	47.8, H	35.0-45.0	%
MCV	103.2, H	80-100	fL
MCH	35.3, H	27.0-33.0	pg
MCHC	34.2	32.0-36.0	g/dL
RDW	8.8	7.5-11.5	fL
ANA screen IFA	NEG	NEG	-
Sm antibody	NEG	NEG	-
Sm/RNP antibody	NEG	NEG	-
Sjögren’s antibody A	NEG	NEG	-
Sjögren’s antibody B	NEG	NEG	-
DNA (ds) antibody	NEG	NEG	-
Westergren sedimentation rate	2	≤20	mm/hour

At the follow-up visit three weeks later, the original lesion was unchanged, but new <1 cm red urticarial papules had developed on the left arm and thigh. Three 4 mm punch biopsies were performed on separate lesions of the arm for histopathology and DIF testing.

**Figure 1:**
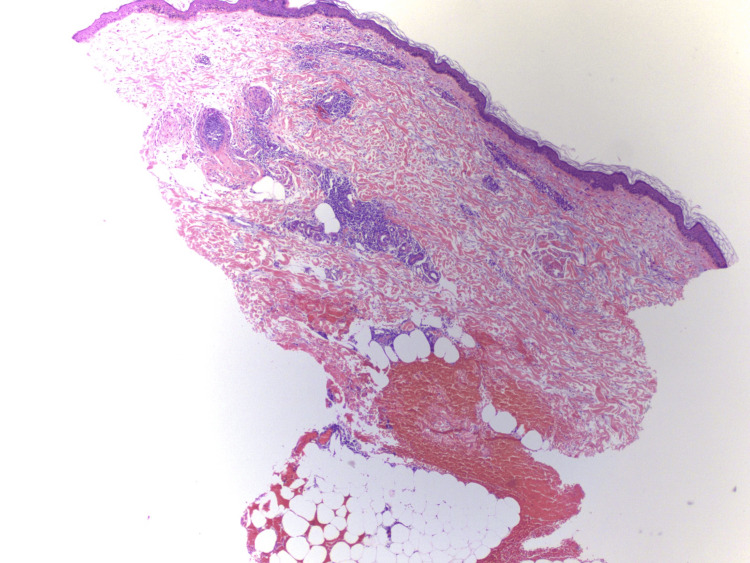
Tumid lupus erythematosus, H&E, original magnification: 5× The bluish hue in the dermis is suggestive of mucin deposition. Lymphocytic infiltrates are prominent. The epidermis appears to be normal H&E: hematoxylin and eosin

All the specimens exhibited superficial and deep perivascular, interstitial, and periappendageal lymphocytic infiltrates. There was a faint bluish hue of the matrix indicative of mucin deposition. Epidermal changes were minimal with a slight thickening of the basement membrane (Figure 1).

**Figure 2:**
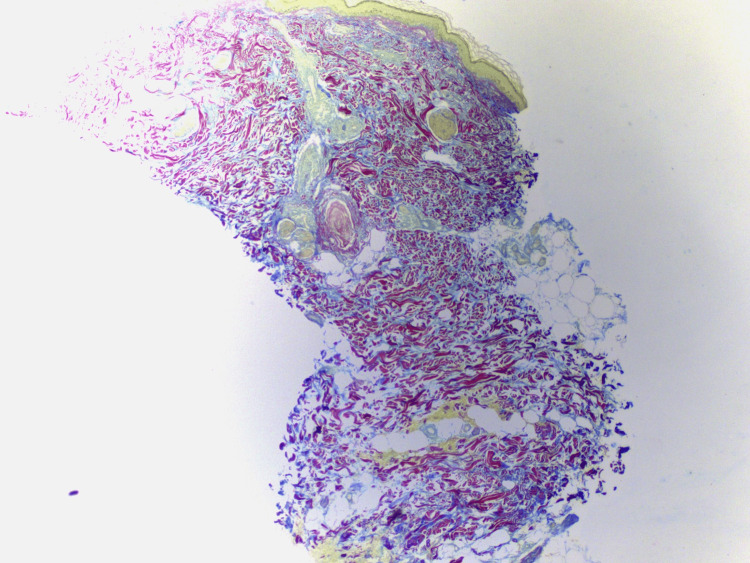
Tumid lupus erythematosus, colloidal iron, original magnification: 5× The blue color between collagen bundles confirms the presence of pools of mucin extending from the papillary dermis to the deep reticular dermis

Colloidal iron stain demonstrated increased superficial and deep dermal mucin deposits (Figure 2).

**Figure 3:**
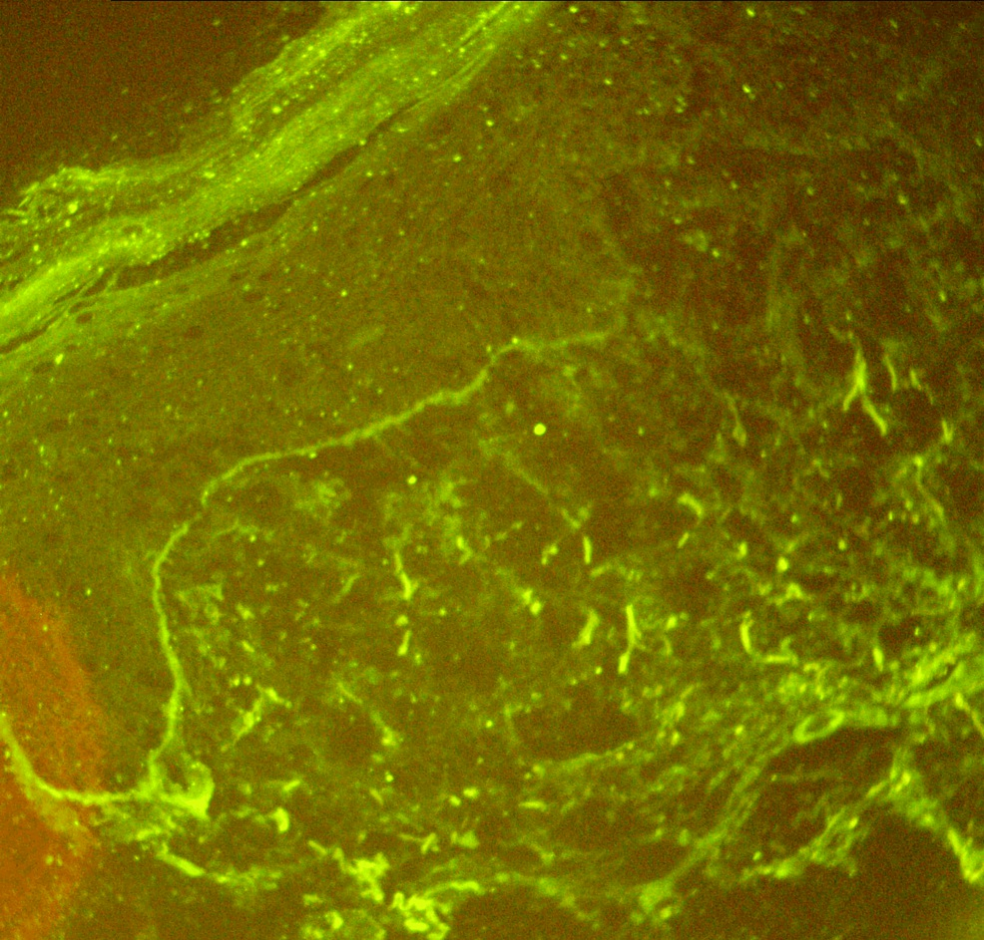
Tumid lupus erythematosus, direct immunofluorescence, IgG, original magnification: 40× DIF demonstrates a linear-granular band of immunoglobulin along the dermo-epidermal junction IgG: immunoglobulin G

DIF revealed a band of IgG deposits along the dermo-epidermal junction (Figure 3).

No other immunoreactants were found. The histopathologic and immunofluorescent findings supported the diagnosis of lupus erythematosus tumidus.

The patient was treated with topical clobetasol cream 0.05% twice daily, advised of the need for sun protection, and reassured that her risk of developing systemic lupus erythematosus (SLE) was low. Hydroxychloroquine therapy was being held in reserve. At the subsequent visit, the LET plaque and papules had resolved after four months without any sequelae such as scarring, atrophy, or dyspigmentation.

## Discussion

Because of the similarities of the clinical lesions, body distribution, photosensitivity, histopathology, and positive therapeutic response to hydroxychloroquine, some authors have considered LET and JLIS to be on a “spectrum” or to be the same entity with different names [11]. While this controversy persisted, Ackerman [12] included LET in his histopathology textbook, which he referred to as “acute discoid lupus erythematosus, tumid type.” In the accompanying photomicrograph, this entity demonstrated orthokeratosis, a lack of follicular plugging, and minimal alterations at the dermo-epidermal interface, compatible with LET.

More recent publications [2,3,13-15] consider LET to be a bona fide subtype of cutaneous lupus erythematosus (CLE) with important obvious differences in clinical appearance, histopathology, immunopathology, autoantibody profile, and prognosis when compared to discoid lupus erythematosus (DLE) and subacute cutaneous lupus erythematosus (SCLE). Although the majority of cases in the largest series published to date [1-3] have had LET as the sole diagnosis, more recent case reports of LET have appeared wherein patients with well-established SLE or DLE and positive serologies have developed LET anew [16]. Dekle et al. [17] reported the case of a patient with the typical changes of LET on initial biopsy who on re-biopsy nine months later showed all the classic features of DLE. In a series of 25 LET cases from Wake Forest University, four patients also had SLE, and one had DLE [1]. Hajji et al. reported the case of a 38-year-old female who presented with biopsy-proven LET and class V lupus nephritis with positive antinuclear, SS-A, and dsDNA antibodies [18]. These cases lend support to the association of LET with other specific subtypes of CLE, as well as SLE.

## Conclusions

LET is an uncommon but separate photosensitive subtype of cutaneous lupus erythematosus that has likely been neglected and underreported. We reported a case of lupus erythematosus tumidus showing a positive IgG deposit at the dermo-epidermal junction by DIF testing and a negative autoantibody profile. LET resolved completely with strict avoidance of sun exposure and topical corticosteroid therapy within four months. Unlike DLE and SCLE, LET may resolve without any sequelae such as scarring, atrophy, or dyspigmentation.

Immunopathologic markers and lupus autoantibodies are infrequently found in LET. Therefore, LET must be differentiated histopathologically from other photosensitive diseases, particularly Jessner’s lymphocytic infiltration of the skin and polymorphic light eruption. The immunohistochemical staining of infiltrating lymphocytes is another method that may help differentiate LET from JLIS and PMLE. CD4 cells predominate in LET, while CD8 cells predominate in the latter two entities. While LET is usually reported as the sole diagnosis, there are case reports of LET occurring in association with DLE and SLE. LET responds well to sun avoidance and protective measures, topical corticosteroids, or hydroxychloroquine.
